# Supplementary material for: Identification of photoperiod-induced specific miRNAs in the adrenal glands of Sunite sheep (Ovis aries)
Source: Front Vet Sci. 2022 Jul 22;9:888207. doi: 10.3389/fvets.2022.888207 (PMC9354845; doi:10.3389/fvets.2022.888207)
Supplement: Supplementary file 1 [file Data_Sheet_1.ZIP › Supplementary file/miRDeep2 file.zip!/miRDeep2 file/Novel_Structure/LP42A1/Novel_422.pdf]

Provisional ID : NC_019460.2_7842
Score total : 2.6
Score for star read(s) : -1.3
Score for read counts : 0
Score for mfe : 2.3
Score for randfold : 1.6
Score for cons. seed :
Total read count : 79
Mature read count : 79
Loop read count : 0
Star read count : 0

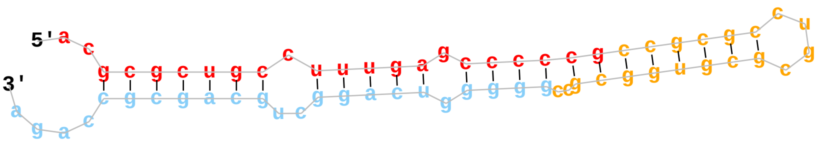

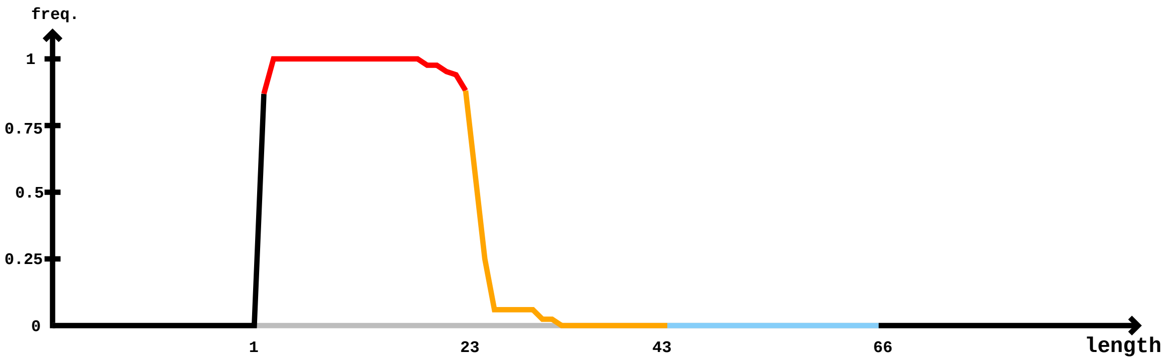

Mature Star

```
5'- ccaaucuaaaugucugaugaacgcgcugccuuugagcccccgccgcgccugcgcguggcgccgggggucaggcugcagcgccagaggacaugcucgcgcauaacuggggguc   -3'    exp
    .........((((((..((...(((((((.(((((.((((((((((((....))))))))..)))).)))))..)))))))))..))))))((((...........)))).    reads    mm    sample
    ....................acgcgcugccuuugagA..........................................................................    1        1     seq
    ....................acgcgcugccuuugagc..........................................................................    1        0     seq
    ....................acgcgcugccuuugagccc........................................................................    2        0     seq
    ....................acgcgcugccuuugagcccc.......................................................................    1        0     seq
    ....................acgcgcugccuuugagccccc......................................................................    5        0     seq
    ....................acgcgcugccuuugagcccccg.....................................................................    41       0     seq
    ....................acgcgcugccuuugagcccccgc....................................................................    6        0     seq
    ....................acgcgcugccuuugagcccccgcc...................................................................    12       0     seq
    ....................acgcgcugccuuugagcccccgccgcgcc..............................................................    2        0     seq
    ....................acgcgcugccuuugagcccccgccgcgccug...........................................................    2        0     seq
    .....................cgcgcugccuuugagcccccg.....................................................................    5        0     seq
    .....................cgcgcugccuuugagcccccgc....................................................................    1        0     seq
    .....................cgcgcugccuuugagcccccgcc...................................................................    4        0     seq
    .....................cgcgcugccuuugagcccccgccgcgcc..............................................................    1        0     seq
```
